# SEE: structured representation of scientific evidence in the biomedical domain using Semantic Web techniques

**DOI:** 10.1186/2041-1480-5-S1-S1

**Published:** 2014-06-03

**Authors:** Christian Bölling, Michael Weidlich, Hermann-Georg Holzhütter

**Affiliations:** 1 Institute of Biochemistry, Charité Universitätsmedizin Berlin, Berlin, Germany; 2 Department of Computer Science, Humboldt-Universität zu Berlin, Berlin, Germany

## Abstract

**Background:**

Accounts of evidence are vital to evaluate and reproduce scientific findings and integrate data on an informed basis. Currently, such accounts are often inadequate, unstandardized and inaccessible for computational knowledge engineering even though computational technologies, among them those of the semantic web, are ever more employed to represent, disseminate and integrate biomedical data and knowledge.

**Results:**

We present SEE (**S**emantic **E**videnc**E**), an RDF/OWL based approach for detailed representation of evidence in terms of the argumentative structure of the supporting background for claims even in complex settings. We derive design principles and identify minimal components for the representation of evidence. We specify the Reasoning and Discourse Ontology (RDO), an OWL representation of the model of scientific claims, their subjects, their provenance and their argumentative relations underlying the SEE approach. We demonstrate the application of SEE and illustrate its design patterns in a case study by providing an expressive account of the evidence for certain claims regarding the isolation of the enzyme glutamine synthetase.

**Conclusions:**

SEE is suited to provide coherent and computationally accessible representations of evidence-related information such as the materials, methods, assumptions, reasoning and information sources used to establish a scientific finding by adopting a consistently claim-based perspective on scientific results and their evidence. SEE allows for extensible evidence representations, in which the level of detail can be adjusted and which can be extended as needed. It supports representation of arbitrary many consecutive layers of interpretation and attribution and different evaluations of the same data. SEE and its underlying model could be a valuable component in a variety of use cases that require careful representation or examination of evidence for data presented on the semantic web or in other formats.

## Background

Scientific evidence, as a concept, can be defined as information that is relevant to assess the likelihood that a particular scientific idea is correct. Representation of the corresponding evidence is therefore key to evaluating hypotheses and assessing claims contained in scientific articles, databases or any other repository of scientific information. Biomedical knowledge is often highly context-dependent and based on evidence obtained from the skilful combination and evaluation of individual results, involving, among other aspects, a range of model organisms, diverse experimental and computational techniques, different forms of interpretation, and various inference schemes. Consequently, all those aspects - the materials, methods and information sources used, the observations made, the reasoning employed and the context-specific assumptions made - are important for comprehensive evidence accounts. Likewise, when data, often from disparate sources, is integrated to study complex biological systems an account of the evidence that was used to infer a model's properties and those of and among its components is critical for correct and transparent understanding of that model.

Scientific findings are now routinely published as resources on the World Wide Web. Besides electronic versions of natural language texts more and more information from both new and legacy sources becomes available through databases [1] and web services [2] which provide through structured formats and interfaces consolidated views of and programmatic access to biomedical data. Semantic web technologies and standards in particular offer by virtue of their well-defined semantics and broad applicability potent means for the computational integration and analysis of biomedical data from heterogeneous and distributed sources on a large scale [3-5]. Accordingly, the Resource Description Framework (RDF, [6]) is increasingly employed to represent and disseminate new and legacy biomedical data [7,8] and biomedical ontologies specified in the Web Ontology Language (OWL, [9]) are being developed to encode domain-specific knowledge and annotate data from biomedical investigations [10-12]. As with any other means for communicating scientific results, findings encoded in semantic web formats need to be accompanied by an account of how they have been established to evaluate their relevance. Towards this end different models, tools and methods have been proposed: for representing and evaluating research hypotheses [13,14], contextualization [15], models of discourse [16], of argument [17], extended means for annotation [18,19], or specific container formats [20]. There is, however, currently no dedicated model supporting a coherent, extensible and semantic-web compatible representation of all those aspects routinely considered by a researcher inspecting the evidence for a given scientific finding, i.e. a representation of (i) the experimental and computational methods and settings that were used to establish the observational results and process the data, (ii) the reasoning including additional findings and assumptions used to infer the result in question, and (iii) information sources and agents through which the corresponding views were communicated and propagated.

Here we introduce SEE (**S**emantic **E**videnc**E**), an RDF/OWL based approach for providing detailed, extensible and computationally accessible accounts of evidence even in complex settings. SEE is designed to enable the fabric of observations, methods, assumptions, and inferences examined by researchers to evaluate the evidence for a claim to be formally represented along with their sources using semantic web techniques. Evidence is captured in terms of the argumentative structure of the supporting background for a claim i.e., by a coherent representation of claims, of the entities the claims are about, of the argumentative relations between the claims and of claim provenance. SEE accommodates nested layers of interpretation and attribution and different evaluations based on the same data. We demonstrate its application in a case study that is typical for the task of collecting, representing and evaluating evidence for systems biology approaches such as genome-scale metabolic network reconstruction by providing an expressive account of evidence for the location of the enzyme glutamine synthetase.

## Results

### Overview of the SEE approach

The SEE approach for representing evidence consists of providing (i) a formal representation of scientific claims, their provenance and the argumentative structure used to justify them by other claims, (ii) a formal representation of claim content and (iii) a coherent integration of the two. SEE relies on an abstract model for the representation of claims, provenance and argumentative structure specified in the Reasoning and Discourse Ontology (RDO), a lightweight OWL vocabulary developed for this purpose. Claim content e.g., *what *is claimed regarding the properties of biological entities or the results and methods of an investigation is represented in RDF graphs by using appropriately defined semantic web resources and design patterns which as a best practice should, if possible, be re-used from existing domain ontologies. The connection between claims as representational primitives and their content relies on named RDF graphs [21] which enable pointing to collections of RDF-triples or OWL-axioms serialized as such.

After outlining general requirements and design principles for representation of evidence we describe the RDO. We then demonstrate the application and design patterns of the SEE approach in a case study generating an expressive representation of evidence reported in the literature for the location of the enzyme glutamine synthetase.

### Deriving design principles and requirements for representation of evidence

We posit two design principles for the representation of evidence and explain their rationale in the following:

DP1: Representation of evidence amounts to representation of claims and argumentative structure.

DP2: Evidence relations in the sense of "A is evidence for B" obtain between the things being claimed.

Accounts of evidence are directed towards the justification of scientific claims. The SEE approach is based on the notion that scientific claims put forward possible, more or less likely scenarios and outcomes - states of affairs [22] - as being accurate descriptions of a subject of scientific inquiry. Something is evidence for a certain state of affairs, if and only if it gives reason to believe that this state of affairs in fact obtains [23]. A pairing of evidence and what it is claimed to be evidence for therefore corresponds to the set of premises and the conclusion of an argument in which the truth of the premises alleges to give reason to believe the conclusion is true. Therefore the evidence used by authors or agents to justify a claim, possibly using further unstated background assumptions, can be mirrored by an argumentative structure having the claim as its conclusion. Typically, what is used to justify the authors conclusions within this argumentative structure are claims in themselves accepted as true on the basis of observations or inferences of the same or of other investigators. SEE, therefore, models evidence relations in the sense of "A is evidence for B" specifically as relations between claims.

We derive two additional requirements:

DP3: A researcher's assessment of the evidence for a finding usually includes evaluation of which materials and methods were used, what kind of data was obtained and which properties were observed, inferred or assumed to establish the finding. Consequently, a representation of the materials, methods, data items and other elements forming the subject of a claim should be part of a computationally accessible evidence representation. In RDF and OWL the subject of a claim, a state of affairs, must be expressed, using appropriately defined resources, as (one or more) triples and axioms, respectively. It follows then, in accordance with DP2 that in an RDF/OWL-based representation of evidence that includes claim subjects the representation of evidential relationships should operate between claim subject representations, i.e. between sets of RDF-triples and/or OWL-axioms.

DP4: Representation of claims and hence representation of evidence must take into account claim provenance, in particular through which source and by which agents the claims were made. Knowing which agent made the claim is crucial for evaluating independence and reproducibility. Tracking the original source of a claim provides a natural reference point for all subsequent representations of the claim and its supporting background and for re-evaluation of the claim within the original context in which it was communicated.

We therefore identify as minimal components for modelling evidence elements representing (i) scientific claims and the argumentative structure used to justify them by other claims, (ii) the subjects of the claims i.e. that what is claimed with regard to a subject of inquiry, (iii) the agents making the claims and arguments, (iv) the sources in which claims were originally made e.g., the original scientific articles or database records.

### Reasoning and Discourse Ontology (RDO)

Based on the foregoing we developed an abstract model for representation of evidence in terms of claims, their argumentative structure and their provenance. It is specified here as the Reasoning and Discourse Ontology (RDO) using the Web Ontology Language (OWL). This section outlines the core classes and properties of RDO. Full, formal specification of all RDO constructs is provided in the ontology file provided as additional file 1.

**Figure 1:**
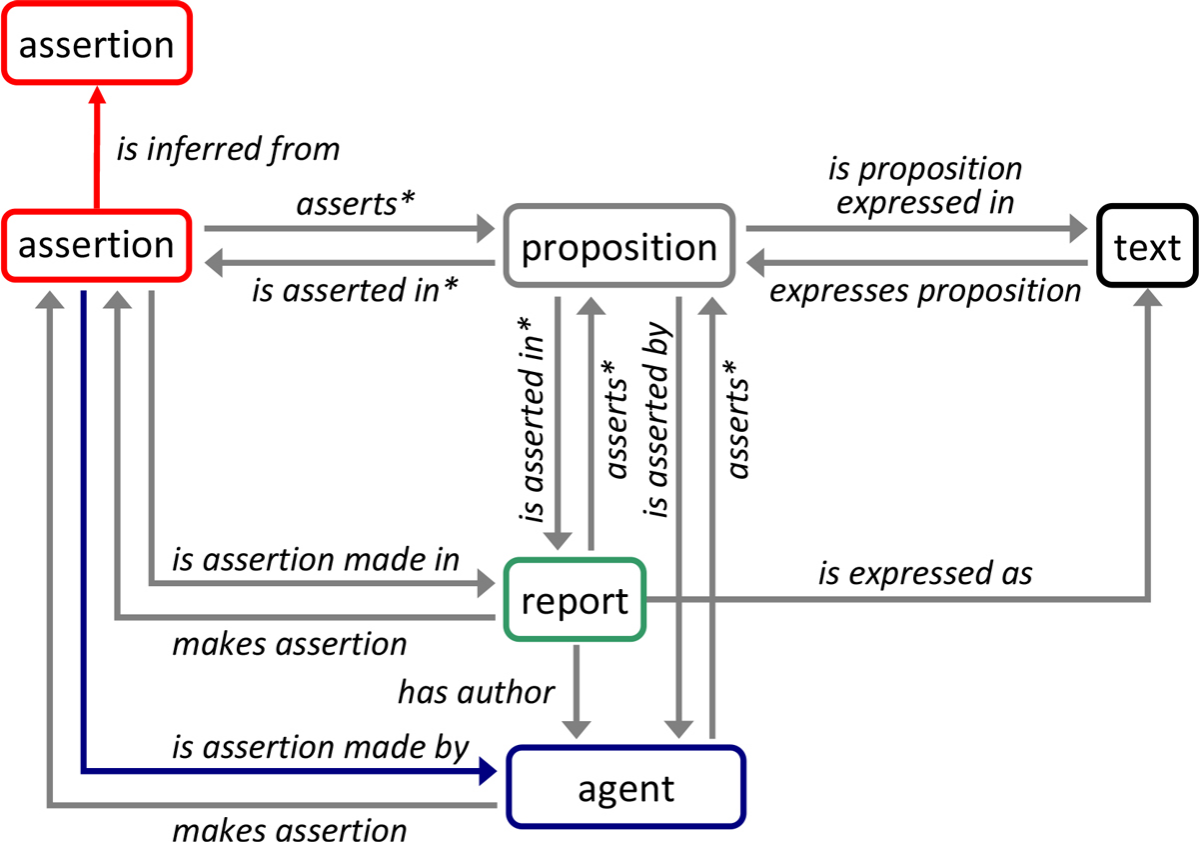
**Core classes and properties in RDO**. Boxes denote labelled classes, arrows denote labelled properties, direction of arrows denotes property domains and ranges. Asterisk: property has domain and range-specific sub-properties. The color code used here is also used in subsequent figures.

The typical scenario that underlies the constructs defined in RDO is the following: Agents (e.g., individual scientists) make claims on particular occasions (e.g., as authors of a published scientific article) about a subject of inquiry. The subject of the claim - i.e. *what *is claimed - is communicated in some linguistic form, often as part of a more comprehensive report (e.g., a scientific article) authored by the agents. Claims are usually justified by other claims the subject of which has been accepted as true, usually on the basis of yet other claims. RDO (Figure 1) rests on the distinction of a claim, its subject and the linguistic form in which this subject is communicated and is centered around the concept of an assertion [24]: instances of the class assertion (courier typeface denotes OWL classes, *courier in italics *denotes OWL properties) represent particular claims made by particular agents on a particular occasion that a particular proposition, the subject of the claim, is true. Propositions, in our model, are represented by the class proposition and taken to represent the semantic content of contextualized lexical entities formulated in some natural or artificial language [25]. The lexical entities by which the subject of a claim and propositions and reports in general are formulated are represented using the class text. Further core classes are report representing accounts intended to accurately describe an event or situation. Thus, scientific journal articles or database records as typical sources of assertions are examples of a rdo:report. Agent is used to represent individual persons, corporate bodies or information processing devices as roleplayers in the creation of reports or assertions. RDO specifies various properties to represent the relations between instances of these classes (Figure 1). In particular, argumentative structure is captured by the property *is inferred from *which relates an instance of assertion to another if and only if the former is, directly or indirectly, inferred from the latter (and possibly other premises).

### Application: representation and evaluation of evidence for a source of glutamine synthetase

#### Introducing the case study

We applied SEE to generate a computationally accessible, expressive and extensible account of evidence gathered in the literature regarding a claimed source of the enzyme glutamine synthetase (GS). We have chosen this particular test case because obtaining reliable information on location of enzyme activities is a subject area of particular importance for systems biology approaches such as the reconstruction of cell-type specific [26] or organism-level [27] metabolic networks. Furthermore, it embodies the typical task of acquiring knowledge on a subject of inquiry by extracting and combining evidence from different sources.

Starting point is our evaluation of a scientific journal article [28] (referred to as '*Meister 1985*' in the following) authored by Alton Meister which asserts in the second paragraph of the text, among other things, that the enzyme glutamine synthetase (GS) was isolated from rat liver. This assertion is based, by way of citation, on the contents of another article by Tate, Leu and Meister [29] (referred to as '*Tate 1972*' in the following). In *Tate 1972 *the isolation of GS from rat liver is reported. The finding is reported to be based on an investigation which involved, among other things, extraction of rat livers, protein purification and γ-glutamyl hydroxamate synthesis (γ-GHS) assays. In the following we show how this context is formalized using the SEE approach to yield a detailed formal account of the evidence presented through these articles for rat liver as source of GS. In doing so, we illustrate various design patterns used in SEE for representing the relevant items. For clarity assertion instances will be indexed as A1, A2, and so forth.

#### Representing the evidence

**Figure 2:**
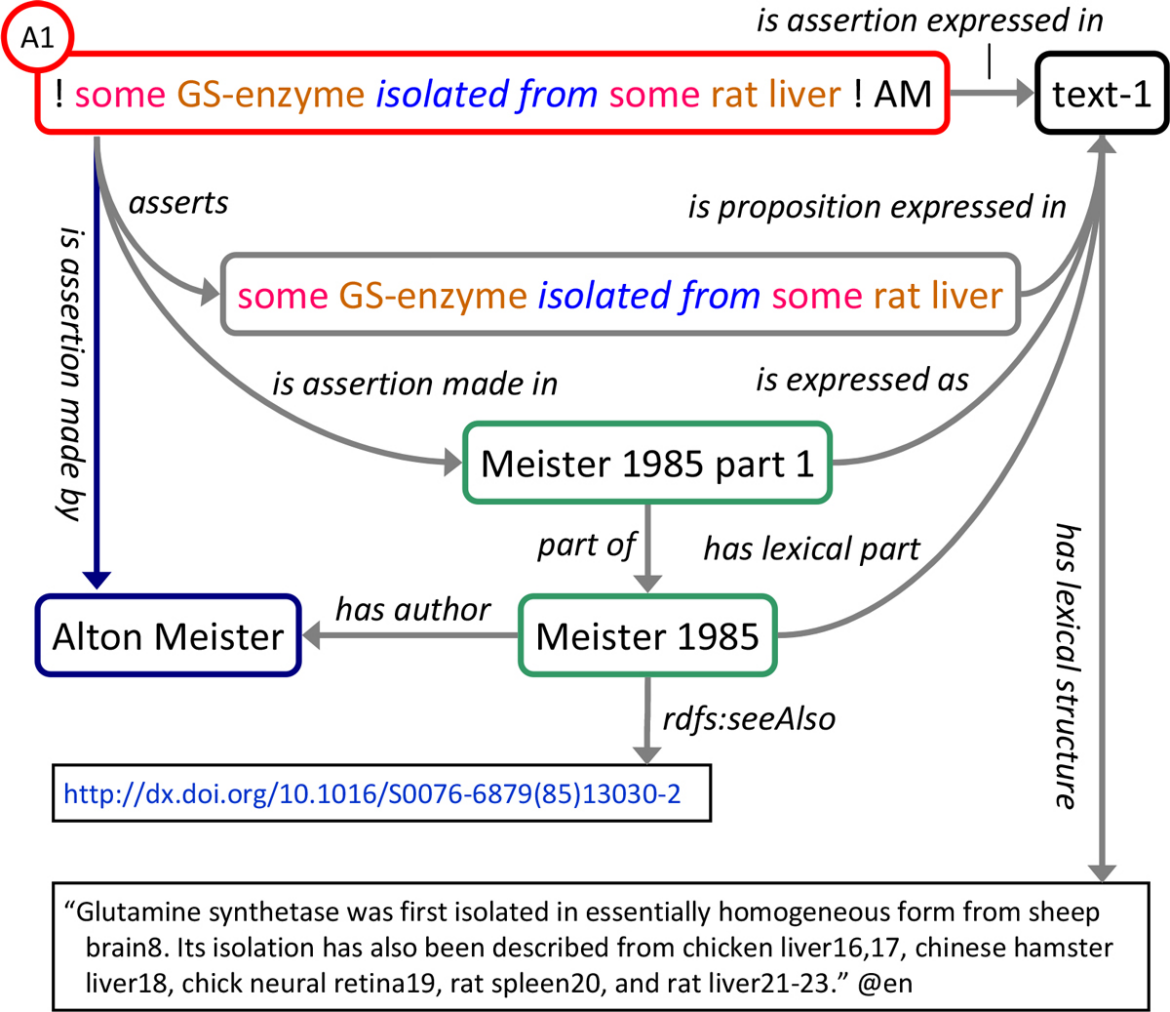
**Representation of assertions with subject and provenance**. Assertion instances are related to proposition instances representing the subject of the assertion by *asserts*, to the agents making the assertion by *is_assertion_made_by *and to the reports in which they are made by is_assertion_made_in. See text for additional relations among assertions, agents, reports and textual representations. Assertion and proposition labels reflect the graph representation of assertion subjects (see text). Color code of assertion and proposition labels indicates the structured representation of assertion subjects (yellow: class, blue italics: property, red: Manchester syntax restriction keyword). Circle shows the index by which the assertion is referred to in the text.

Figure 2 shows how the assertion from *Meister 1985 *that GS was isolated from rat liver is represented using RDO, exemplifying the design pattern used to represent the relations between a particular assertion and its subject and provenance: The article itself, *Meister 1985*, is classified as instance of report annotated with a uniform resource locator (URL) providing its digital representation. The second paragraph of *Meister 1985 *constitutes a report_part. It is expressed as the English language text as which it is written and which is represented as an instance of text. The original text is linked to it via the data property has_lexical_structure. Meister's claim that glutamine synthetase was isolated from rat liver contained in this paragraph is represented by an instance of assertion (A1) labelled as '! some GS-enzyme isolated from some rat liver ! AM' to indicate the assertion subject in a concise, human readable manner (formalization of assertion subjects is described below). A1 is related to a corresponding instance of proposition identifying the subject of the claim, to an instance of agent representing Alton Meister, and to said report part by the properties *asserts, is_assertion_made_by *and *is_assertion_made_in*, respectively.

**Figure 3:**
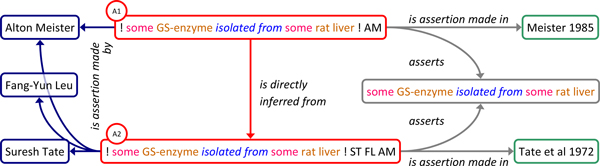
**Representation of claims which reiterate previous findings**. The fact that Meisters assertion (A1) reiterates what Tate & co-workers have asserted on the isolation of GS from rat liver (A2), is represented by a relation of the former to the latter via *is_directly_inferred_from *and by sharing the same proposition instance via *asserts*. Each assertion instance is linked to its corresponding agents and reports. Color code as in figure 1.

Claims which reiterate previous findings are represented as assertions on the same subject made by the respective agents. Formally, the reiterating claim is represented as an assertion instance which is linked to the source assertions by *is_directly_inferred_from *and linked to the same proposition instance as the source assertions by *asserts*. Each assertion can be linked to its corresponding agents and reports. Application of this design pattern to our case study is shown in Figure 3: The fact that Meisters assertion (A1) reiterates what Tate & co-workers have asserted on the isolation of GS from rat liver (A2), is represented by a relation of the former to the latter via *is_directly_inferred_from *and by sharing the same proposition instance via *asserts*.

**Figure 4:**
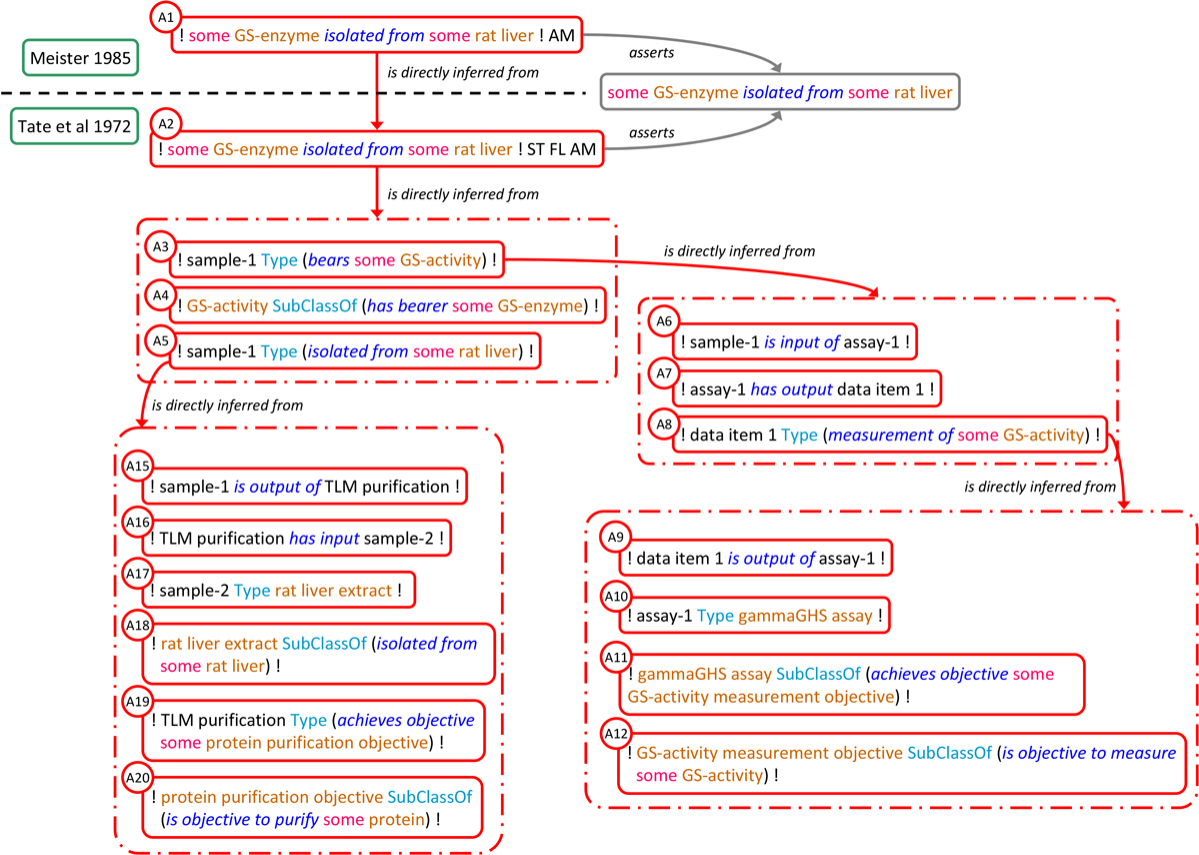
**Representation of argumentative structure within and across publications**. The argumentative structure used to justify Meister's claim on the isolation of GS from rat liver (A1) is represented as a series of assertion instances linked by *is_directly inferred_from *relations. Dashed line separates assertions made in *Meister 1985 *and *Tate 1972*. Dashed-dot boxes indicate composite assertions with their component assertions placed inside signifying the *has_conjunctive_part *relations. Author initials tags of the assertions made by Tate et al. are omitted, as are links to propositions, reports, authors and texts. Color code as in figure 1.

The argumentative structure within and across the publications is represented as a series of assertion instances and *is_directly_inferred_from *relations with additional links to represent assertion subjects and provenance (Figure 4). The assertion instances linked to A2 reflect the results and the reasoning of the authors at various steps of their investigation based on a careful analysis of the internal argumentative structure of *Tate 1972*. Specifically, Tate et al.'s main conclusion that GS-enzyme was isolated from rat liver (A2) is essentially based on asserting that (A3) there is a biological sample (labelled 'sample-1') which has GS-activity, that (A4) any GS-activity is borne by some GS-enzyme and that (A5) sample-1 was isolated from some rat liver (precise definitions for GS-enzyme, GS-activity in the context of the case study are detailed in additional file 2). The joint use of A3, A4 and A5 to infer A2 is made explicit by using the *has_conjunctive_part *property to link them to the same composite assertion instance which in turn is related to A2 using the *is_directly_inferred_from *property. This pattern is used whenever an assertion is inferred from more than one premise. A3, the assertion that sample-1 has GS-activity is justified in turn by asserting that (A6) it was input to a particular assay (labelled assay-1), that (A7) this assay produced a particular result, data item 1, and that (A8) this data item is a measurement of some GS-activity. A8, in turn, is justified by asserting that (A9) the data item is output of assay-1, that (A10) this assay was a γ-GHS assay, and that (A11 & A12) this type of assay is suited to measure GS-activity. Some assertions are not further justified, either because they reflect factual descriptions in *Tate 1972 *(A9, A10), represent general assumptions of the authors (A11) or are expressions of terminological domain knowledge (A12, A4). A5 exhibits a similar justification trail, as shown in Figure 4. Full, formal representation of the argumentative structure for the test case is provided in additional file 2.

The prevalent pattern in SEE for recording individual and logically relevant steps of an investigation is for any such step to link its outcomes (data or material), the techniques used to produce these outcomes, and their objectives as exemplified in the composite assertions comprising assertions A9-A12 and A15-A20 (Figure 4). In A9-A12, for example, the experimental process type (γ-GHS assay) is linked to the objective of its application (GS-activity measurement) and in turn to the quality that is intended to be determined (GS-activity). Generally, the relations between these ontologically different entities are not trivial and not one-to-one (one objective can consist of the determination of several qualities recognized in a scientific domain, a certain quality can be the subject of inquiry in several objectives). However, in this particular case the objective and quality are narrowly defined and directly correlated.

#### Representation of assertion subjects

**Figure 5:**
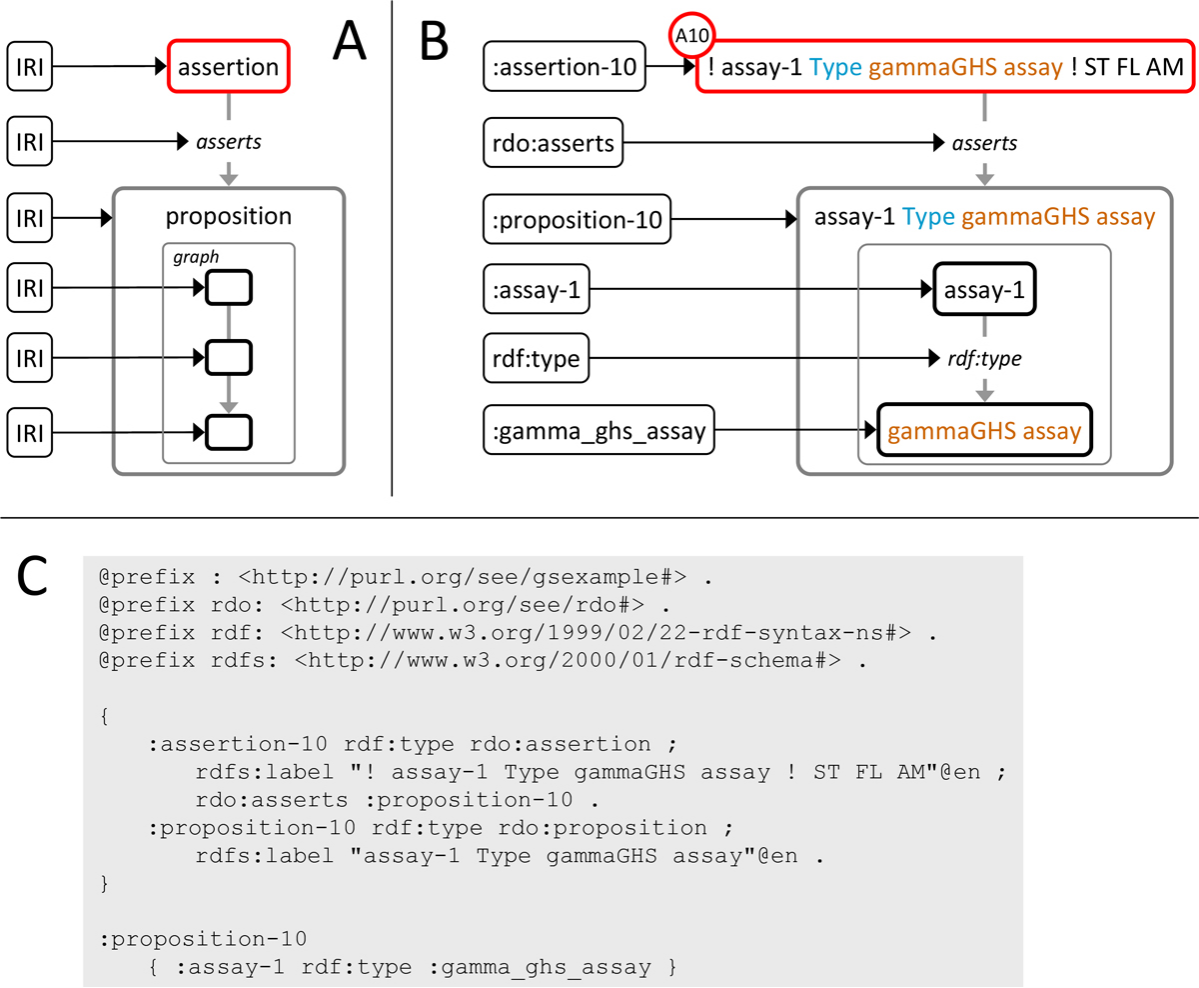
**Representation of assertion subjects as named graphs**. In SEE structured, queryable representations of assertion subjects are provided as named graphs. A) The structured representation of the subject of an assertion can be accessed as the RDF graph identified by the IRI of the proposition instance related by the *asserts *property to the assertion instance representing the assertion. B) Structured representation of the subject of assertion A10 asserting that the particular assay performed by Tate et al. (:assay-1) was a γ-GHS assay (:gamma_ghs_assay). C) TriG representation of the graph shown in B.

The representation of argumentative structure and claim provenance as an interrelated set of assertion instances described so far is complemented by a structured representation of *what *is asserted in each assertion, the assertion subject. To this end each assertion instance is linked to a corresponding proposition instance the IRI (Internationalized Resource Identifier) of which identifies a named RDF graph. This graph provides a structured representation of the assertion subject using appropriately defined resources (Figure 5). This setup enables querying the elements forming the assertion subject. In assertion A10, shown in Figure 5 as an example, it is asserted by Tate and co-workers that the particular assay they performed was a γ-GHS assay. The representation of this statement as a graph identified by the IRI of the proposition instance linked to the assertion instance representing A10 enables to access the entities A10 is about: the particular assay, its asserted type, and the typing relation itself. Full specification of all propositions as named graphs in the context of the case study is provided in additional file 3.

To generate the graph representations of the assertion subjects, the natural language expressions of the assertions identified in the *Meister 1985 *and *Tate 1972 *reports were formalized in RDF using appropriately defined resources (see additional files 2, 3 and 4). Most assertion subjects could be formalized in a straightforward manner applying OWL 2 RDF-based semantics [30]. The principal claim that "glutamine synthetase was isolated from rat liver" which is the common subject of assertions A1 and A2 was formalized in RDF by instantiating the class gs_enzyme and *is_isolated_from *some rat_liver (shown as :proposition-1 in additional file 3). This exemplifies instantiation of the OWL-class (A and *related_to *some B) as a design pattern for formalization of statements which can, in natural language, be represented in the form "some A related to some B" (A and B denoting OWL-classes used to represent the types A and B, respectively and *related_to *denoting an OWL-property used to represent the relation among some of their instances).

Labels of assertion and corresponding proposition instances are directly derived from the graph representation of the assertion subject (see methods section). In particular, the label "some A *related_to *some B" is used for proposition instances that represent statements of the form "some A related to some B" by applying the design pattern described above.

#### Representing consecutive layers of interpretation and own conclusions

**Figure 6:**
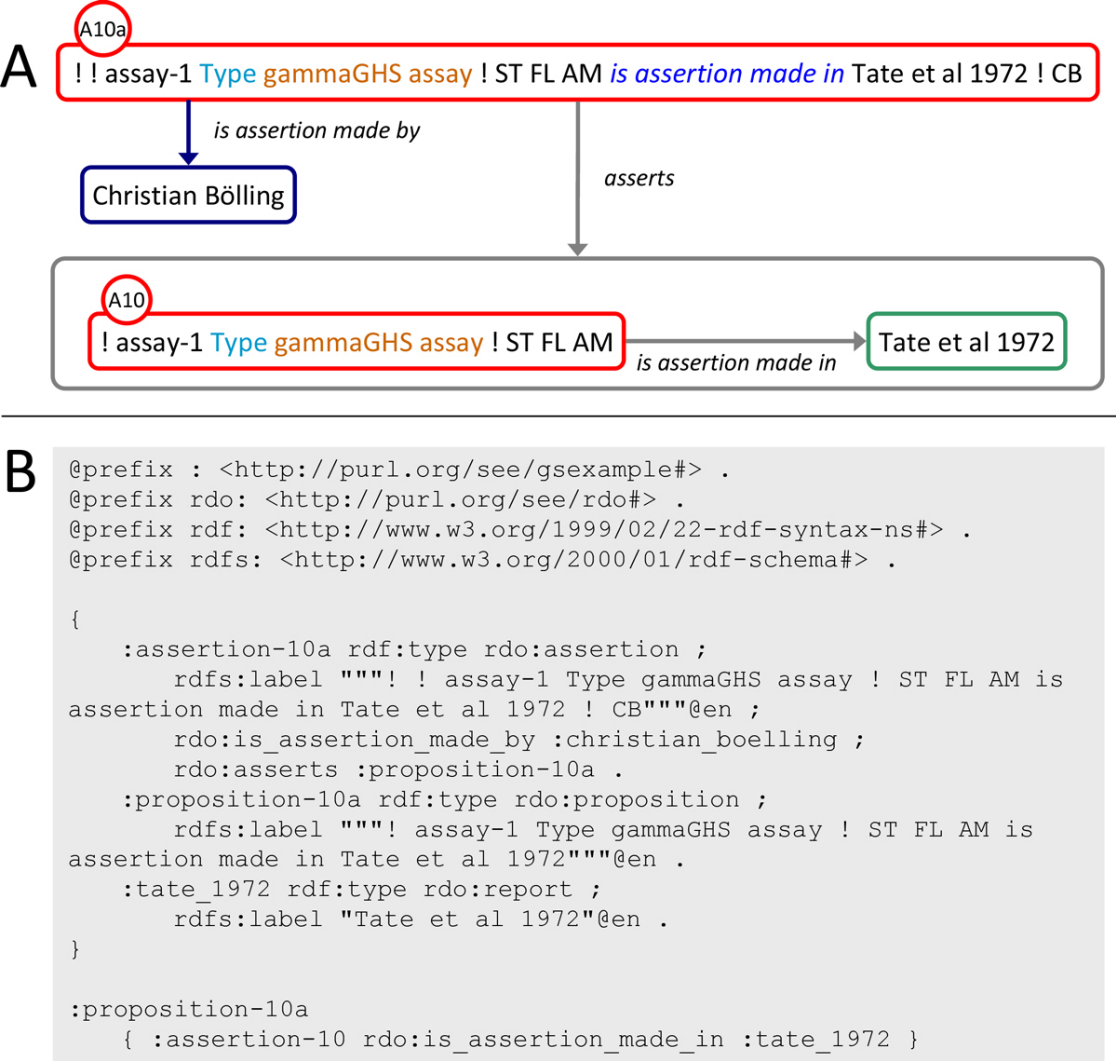
**Representation of consecutive layers of interpretation**. Consecutive layers of interpretation can be represented as assertions the subject of which is about other assertions. A) CB's assertion that 'Tate and co-workers assert that assay-1 was a gamma-GHS assay in their 1972 report' is represented as an assertion instance linked to a proposition instance whose named graph representation relates the assertion instance A10 to the *Tate 1972 * report via the *is_assertion_made_in *property. B) TriG representation of the graph shown in A. In combination with the RDF dataset shown in Figure 5C this is an example of a named graph referencing a named graph via the corresponding assertion and proposition instances. Color code as in figure 1.

We use the test case to specify additional design patterns to represent activity of a curator or generally of a third party evaluating a scientific report. Our representation of the evidence in the *Meister 1985 *and *Tate 1972 *reports is the result of the interpretation by another agent (Christian Bölling - CB). This can be explicitly represented in SEE using its familiar design pattern for propositions and assertions. For example, the claim that Tate et al. indeed assert that the assay they performed was a γ-GHS assay in their 1972 publication can be represented as an assertion instance in its own right, made by another agent, CB (Figure 6). This pattern allows for representing arbitrary many consecutive layers of interpretation or attribution.

**Figure 7:**
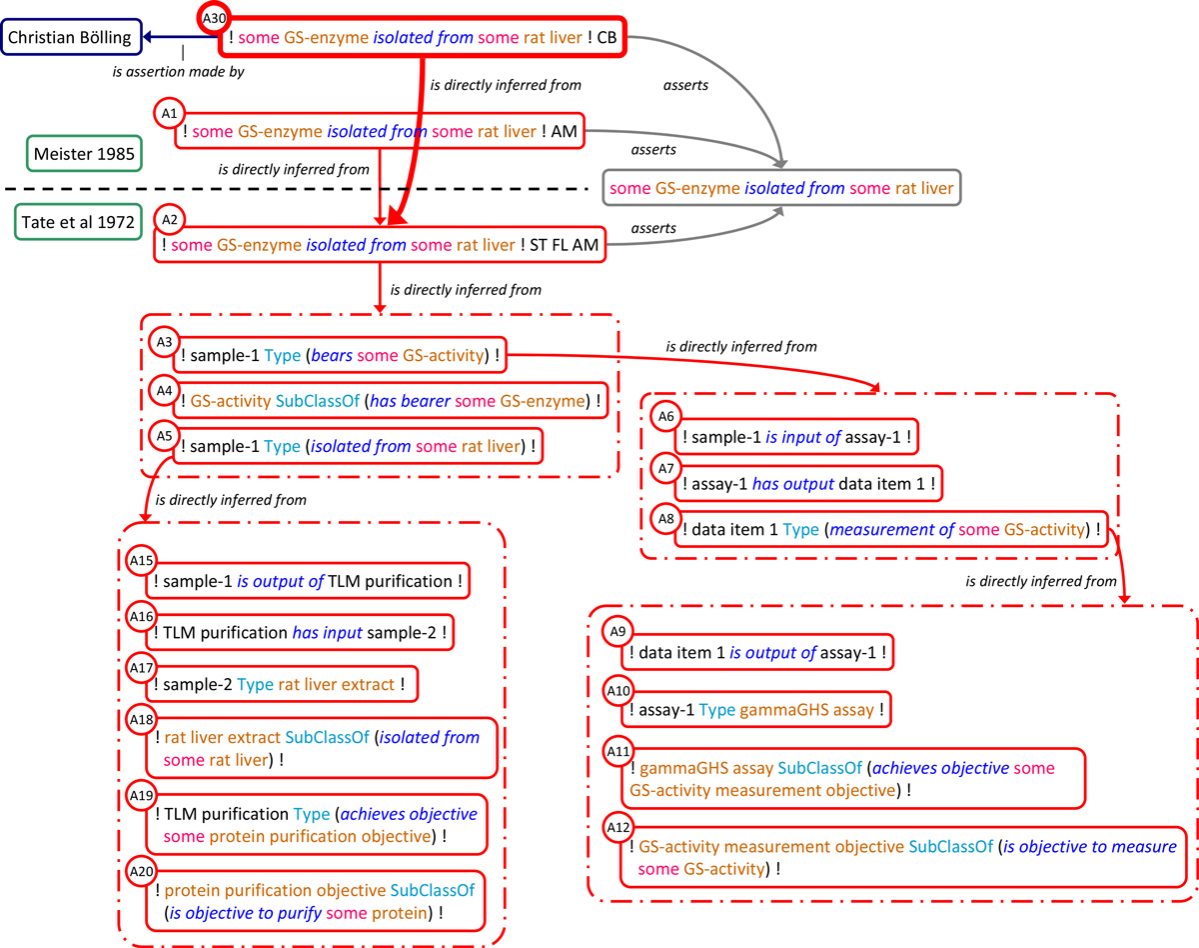
**Representation of curator activity: inference from author statement**. The pattern to represent inference from author statement is illustrated here by linking curator assertion A30 (shown in bold) to assertion A2 of the Tate et al. argumentation signifying inference of A30 on the basis of an author statement of Tate et al. on the same subject. Color code as in Figure 1.

**Figure 8:**
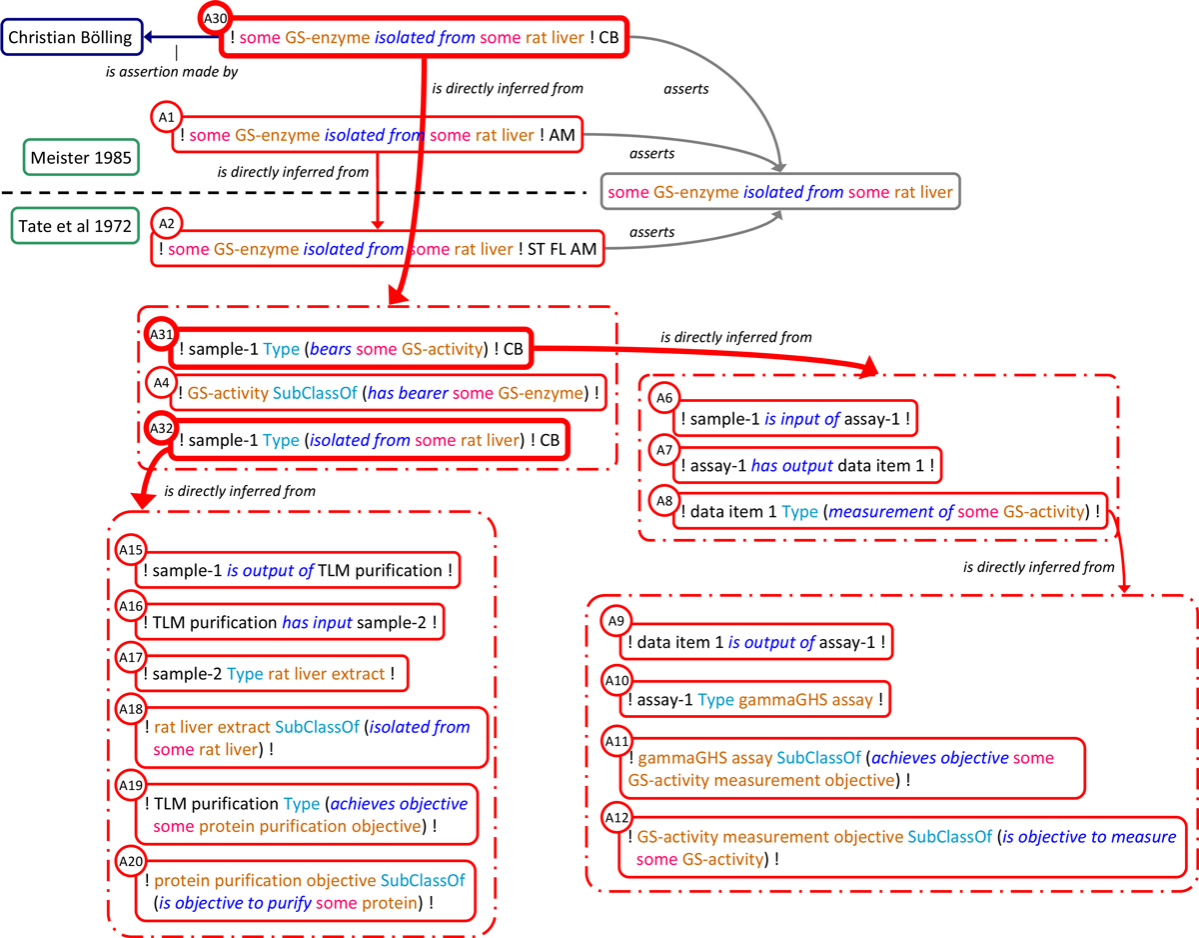
**Representation of curator activity: inference from experimental evidence**. The pattern to represent inference from evaluation of the reported experimental evidence, in contrast to inference based on author statement (Figure 7), is illustrated here by linking curator assertion A30 to a new composite assertion involving curator assertions A31 and A32. These are, in turn, linked to the composite assertions A9-A12 and A15-A20, respectively. These composite assertions reflect data and procedures reported in *Tate 1972*. Taken together this graph therefore represents a curator conclusion (A30) based on the affirmative outcome (A31, A32) of the evaluation of the data and procedures reported in *Tate 1972*. Note that A2, the principal conclusion of Tate et al. is unrelated to the new composite assertion. Curator assertions and their links to the Tate et al. argumentation are shown in bold. Color code as in Figure 1.

So far the presented account consists of assertions attributed to the authors of the *Meister 1985 *and *Tate 1972 *reports, i.e. a representation of what these authors assert. SEE also provides the resources to append own conclusions. For example, an agent, CB, could upon evaluation of the claims made by Tate et al. conclude for *himself *that GS was indeed isolated from rat liver. This is represented as an assertion instance in its own right (A30, labelled '! some GS-enzyme isolated from some rat liver ! CB'). It is linked to the corresponding proposition via *asserts *and the assertions made by Tate et al. via *is_directly_inferred_from*. We describe two semantically different patterns to make this connection. In pattern 1 assertion A30 is linked to assertion A2 (Figure 7). In pattern 2 (Figure 8) A30 is linked to a new composite assertion that involves two more curator assertions (A31, A32) and A4 as a representation of terminological domain knowledge. A31 and A32 are linked by *is_directly_inferred_from *to composite assertions reflecting factual descriptions of data and procedures given in *Tate 1972*. There is a subtle, yet important difference in meaning between these two representations. In pattern 1 CB's conclusion is based on Tate et al.'s assertion on the same subject, i.e., it is based on the author statement itself and does not necessarily imply an affirmation of how Tate et al. reached their conclusion. In pattern 2 the curator inference is based on factual descriptions in *Tate 1972*, i.e., it affirms the conclusions of Tate et al. as own conclusions on the basis of the reported experimental results.

**Figure 9:**
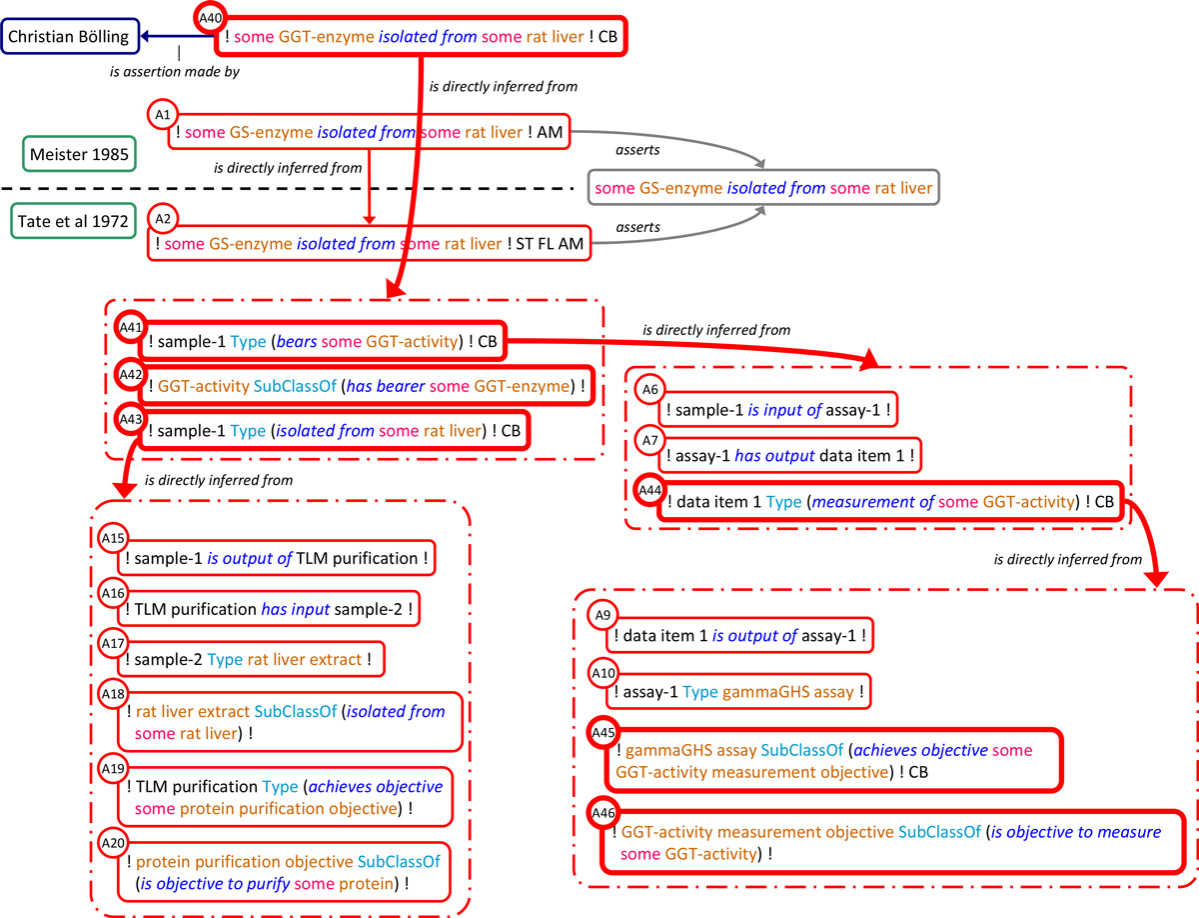
**Representation of curator activity: alternative interpretations of reported data**. Alternative interpretations of reported data can be represented as assertions that are made by a third party and linked to assertions reflecting factual descriptions of the reported data. Based on CB's assertion that γ-GHS assays measure GGT activity (A45) - rather than GS-activity - it is inferred that GGT-enzyme has been isolated from rat liver (A40). The corresponding inference chain relies on a number of new curator assertions (A41-A46) and their combination into composite assertions but re-uses assertions on the quantitative data obtained and the procedures conducted by Tate et al. Curator assertions and new inference links are shown in bold. Color code as in Figure 1.

Evaluation of a given set of data might also lead to conclusions different from those of the authors. Such alternative interpretations can be represented using SEE. For example, one might dispute that γ-GHS assays are suited to measure GS-activity (EC 6.3.1.2). The γ-GHS assay works by measuring the formation of L-γ-glutamyl hydroxamate rather than glutamine [31]. Tate et al. assert as the objective of its application GS-activity measurement, accepting the formation of the hydroxamate under the conditions of the assay as a proxy for the formation of glutamine and the actual reaction mechanism. Assertion A11 using the property *achieves_objective *reflects this acceptance by Tate et al.. Alternatively, a third party could assert that γ-GHS assays merely achieve the less specific objective of measuring γ-glutamyl transferase (GGT) activity (EC 2.3.2.2) (Figure 9, assertion A45). In this case the data reported by Tate et al. can still be used to infer that rat liver is a source of GGT-enzyme (Figure 9, assertion A40).

#### Evaluating the test case evidence representation

The test case evidence representation that was created using the RDO constructs and design patterns was evaluated in terms of its potential to answer, within the confines of the case study, a list of competency questions reflecting different aspects of the evidence a researcher investigating glutamine synthetase knowledge would be interested in:

Q1: Which locations of GS have been asserted?

Rat liver.

Q2: Where has rat liver GS been reported?

The Meister 1985 and Tate 1972 reports.

Q3: Do the assertions made in these reports pertain to independent observations?

No. Meister's assertion is based on Tate et al.'s assertion. Moreover, some of the authors of the two reports are identical.

Q4: Is there experimental evidence and where is it described?

Yes. In the Tate 1972 report.

Q5: Which observations and techniques were used for establishing rat liver as GS source?

1. extraction of a protein sample from rat liver (technique: TLM purification)

2. that sample has GS-activity (technique: γ-GHS assay)

Q6: Did Tate et al. really make these observations and conclusions? Who created this account of their findings?

Christian Bölling.

**Figure 10:**
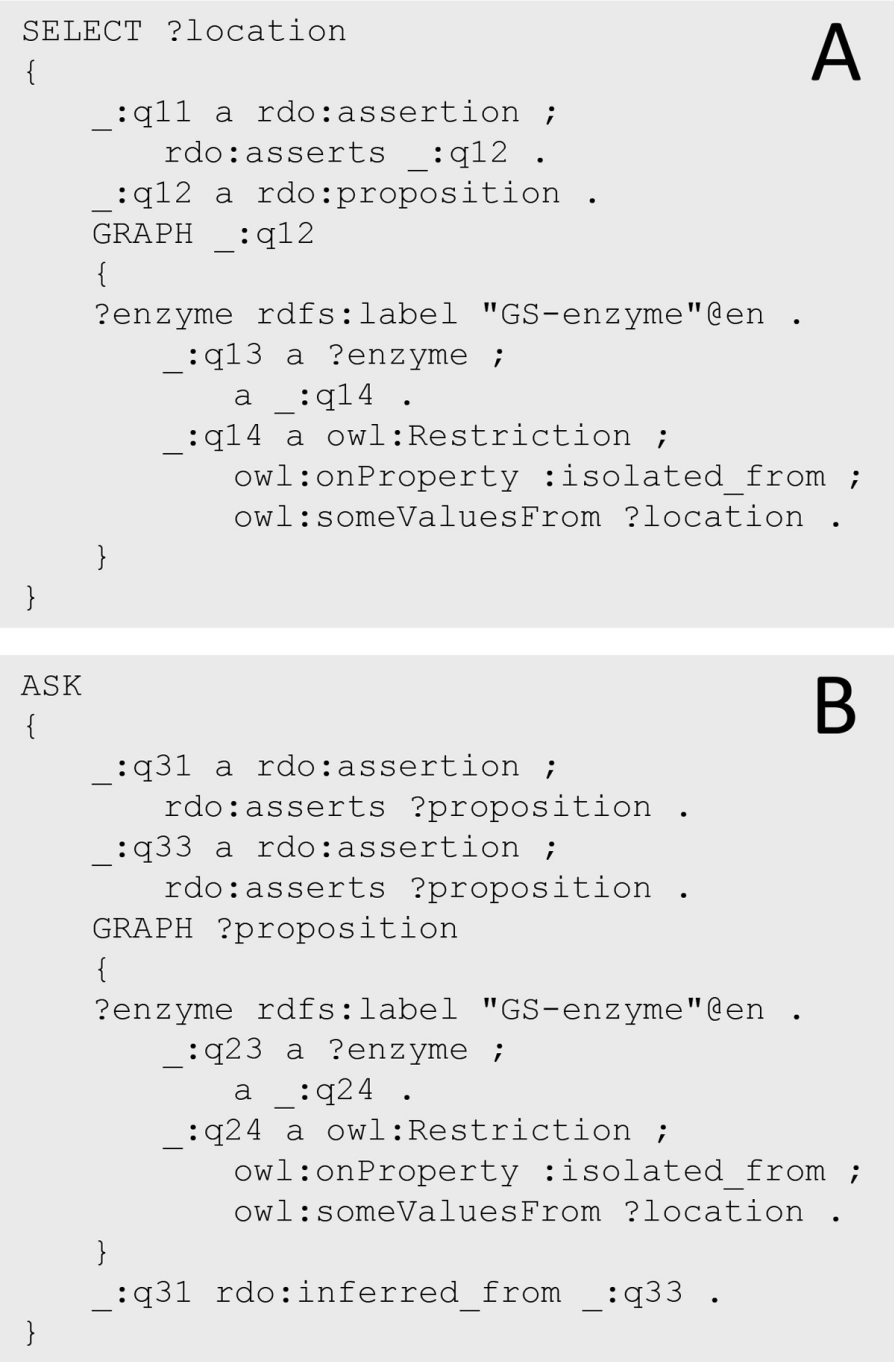
**Competency questions SPARQL queries**. A) SPARQL query to identify all asserted locations of GS (Q1). This query identifies patterns in which an assertion (_:q11) has a subject (_:q12) which includes a graph pattern indicative for the isolation of GS from some location. B) SPARQL query to identify dependency of assertions on the same subject. The query identifies assertions (_:q31, _:q33) which share the same subject (?proposition) and are inferred from one another.

Based on the SEE design patterns, these questions could be formulated as SPARQL [32] queries and successfully answered (see additional file 5). In each of Q1-Q6 the structured representation of assertion subjects as named graphs, besides the other SEE design patterns, is used to identify assertions which are relevant to answer the query. For answering Q1 assertions are identified whose subject's graph representation includes a graph pattern indicative for the isolation of GS from some location (Figure 10A). For answering Q3, pairs of assertions are identified whose subjects share the same graph representation and where one is inferred from the other (Figure 10B).

The following evidence-related information can be queried exploiting property chains and other axioms defined for the RDO constructs:

- all assertions which are directly or indirectly used to infer a given assertion

- all assertions made in a given report

- all assertions made by a given agent

- all assertions on the same subject

- all agents making assertions on a given subject

**Figure 11:**
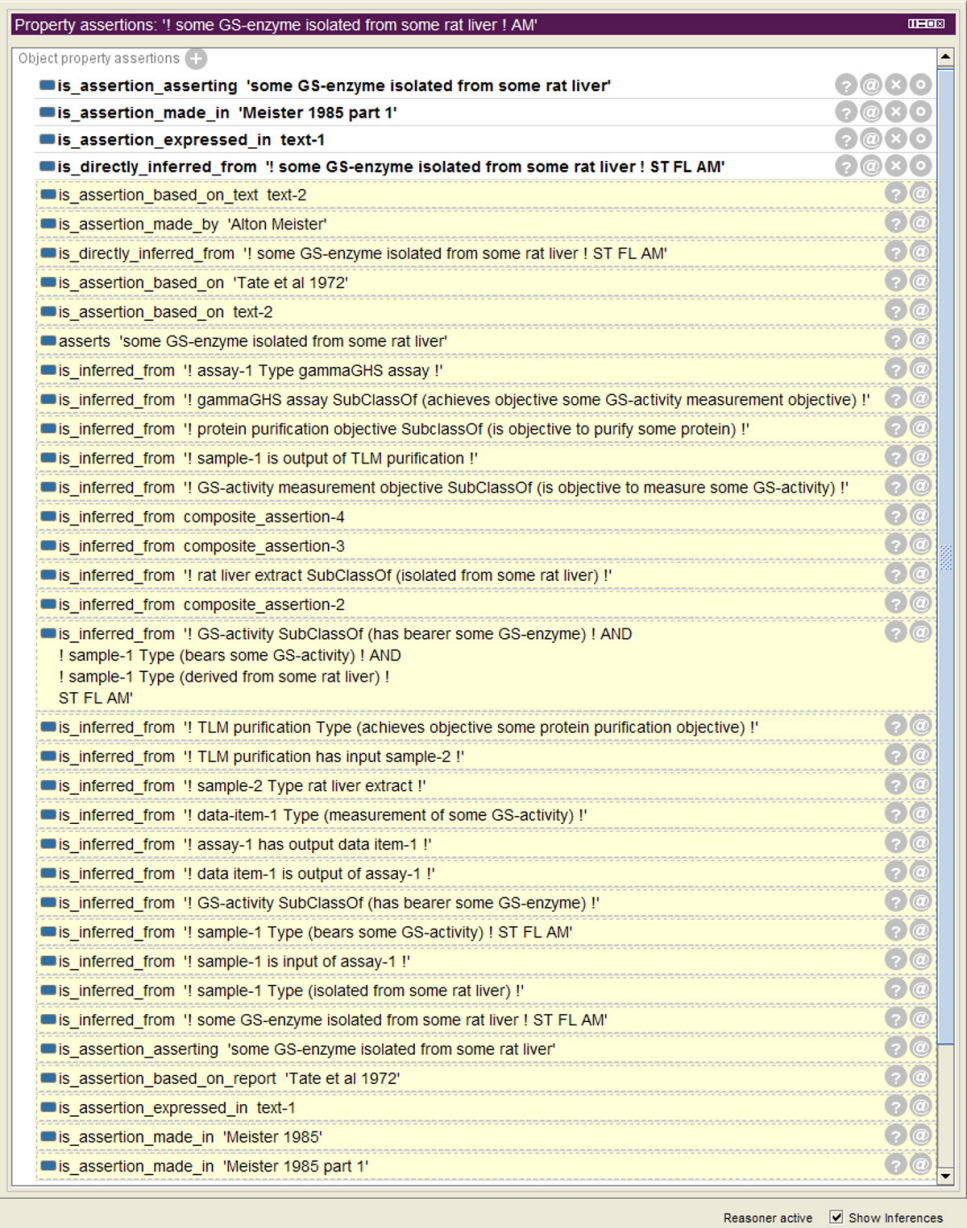
**Inferred object property assertions**. Inferred object property assertions for Meister's assertion that GS was isolated from rat liver (A1).

For the corresponding queries see additional file 5. As an example, in Figure 11 the object property assertions inferred for assertion A1, Meister's assertion that GS was isolated from rat liver, are shown. These inferences, simply derived in Protégé 4 with HermiT 1.3.8 as a reasoner include all assertions which A1 is directly or indirectly inferred from and all reports and texts A1 is based on.

## Discussion

### SEE design

SEE offers a tangible interpretation of the concept of evidence in terms of the argumentative structure of the supporting background for a claim. It rests on the disctinction between claims as such (assertion), their subjects (proposition) and the linguistic form in which these subjects are communicated (text). As a consequence of this design evidential relations (as in "A is evidence for B") can be represented consistently as relations between assertions. This means that statements of the form "this dataset / experiment / publication / method is evidence for B" are regarded as figurative expressions. Instead, the relation between a dataset, an experiment or a publication and the state of affairs it is claimed to be evidence for is represented indirectly through relations between assertions the subjects of which relate to the entities in question. The advantage of this design is that it enables a coherent representation not only of extensive argumentative networks but also of arbitrary many layers of consecutive interpretations and alternative evaluations of the same observations or information sources. RDO offers clear, formally defined types and relations for representing claims, their subjects, their linguistic representations, related information sources and agents on the basis of well established concepts from epistemology and the philosophy of language [22,24,25,33,34]. The case study examples suggest that the SEE design principles and their implementation in RDO are capable of correctly representing, in a computationally accessible and coherent form, the entire 'evidence trail' for a claim needed to evaluate its relevance including observational data, research techniques, assumptions and information sources.

SEE represents argumentative structure at its foundational level of premises being used to infer a conclusion using the *is_directly_inferred_from *property and its transitive superproperty *is_inferred_from*. This allows for a coherent representation of different argument forms and larger rhetorical structures which can be mapped onto their underlying assertions.

SEE aims to capture arguments as they are presented in their sources rather than to evaluate their quality or to categorize them. How conclusive an argument is will typically depend on agent background knowledge or application-dependent requirements. The SEE design enables users to evaluate evidence according to their own, possibly domain- and application-specific criteria.

SEE-based accounts could also be used alongside specified rules, or argument forms considered as acceptable by individual researchers or within specific domains of inquiry which could then be leveraged to automatically infer new assertions on the basis of the already asserted information.

With regard to the extraction of assertion subjects and a specific argumentative structure from a natural language text SEE relies in its current form on a heuristic approach leveraging expert domain knowledge to identify assertions and formalize them in OWL. As OWL is a subset of first order logic there may be statements from natural or artificial languages which cannot directly be translated into OWL, constraining the formalization of assertion subjects in SEE. It is, however, not clear which actual limitations arise from this theoretical constraint for the representation of evidence in specific use cases. The test case presented here suggests that within a specified domain of discourse, using appropriate constructs and design patterns, the relevant contents of the statements made originally in a context-rich narrative format such as a scientific journal article can be adequately formalized.

Formalization of natural language statements is an important prerequisite for computational approaches to data evaluation. For applications that can forego this need the statements can be represented in their original form as texts or referenced by links to the original information sources. Both are by default designed to be provided in SEE as reference points for evaluation.

The presented design patterns make SEE-based accounts of evidence extensible. This design is in line with the open world assumption on which RDF and OWL as knowledge representation languages operate. The particular argumentative structure and level of detail presented in the case study are based on heuristics reflecting domain-specific requirements to understand how an enzyme was characterised. This representation can be extended or shortened as required. For example, details on the protein purification process performed by Tate et al. or indeed any other detail that becomes relevant for the evaluation of the presented evidence could be appended to the existing assertions. Likewise, as we have demonstrated, alternative views and conclusions can be accommodated. On the other hand, for applications which only require information on claim provenance, only the source publications of the main claims could be represented.

### Evidence types

Evidence type schemes provide a useful shorthand categorization of research techniques used to establish a claim. SEE could be aligned with any categorization of research techniques and hence evidence type scheme to characterise the evidence for an assertion. Essentially, SEE provides a platform to define custom, extensible evidence types and apply them as needed. For example, the evidence for rat liver as a source of GS in the test case could be characterised as "experimental evidence" as "based on a direct assay" as "based on a γ-GHS-assay" or as "based on a γ-GHS assay, protein purification involving Sephadex chromatography, and samples from Sprague-Dawley rats" depending on the level of accuracy desired.

The flexibility and extensibility of the SEE approach may also be useful to characterise evidence where several techniques have been combined to establish a scientific result or evidence is characterised in combination with claim provenance. We illustrate this with a comparison to the Gene Ontology (GO) evidence codes which are meant to reflect the type of work or analysis described in the cited reference which supports the GO term to gene product association [35]. GO evidence codes consist of a collection of terms arranged in a hierarchical format. In this taxonomy the terms representing justifications based on author statements (TAS, NAS) are unrelated to those representing experimental techniques (EXP and child terms). Consequently, GO associations marked as being made on the basis of an author statement are usually not qualified with respect to how this author statement came about. In contrast, as demonstrated in the case study, using SEE any author statement can be extensively qualified in terms of the experimental evidence or other author statements it is directly or indirectly based on.

### Use cases

Representations which use SEE or its underlying model could be productive in a variety of use cases requiring careful examination or recording of evidence, e.g.,

- providing supporting background information for biomedical knowledge bases,

- creating digital abstracts of research publications,

- adding a claim-level perspective on research publications which could be used by publishers, in bibliographic databases and in personal bibliography managers,

- providing open linked data which can be integrated on an informed basis using varying, application specific evidence criteria.

### Related and future work

The SWAN biomedical discourse ontology [16] developed in the context of the Semantic Web Applications in Neuromedicine (SWAN) project offers a formal model of scientific discourse based on two different classes of statements; swan:hypothesis and swan:claim. Claim subjects are to be represented in natural language and the resolution of their supporting background is confined to the document level. The Annotation Ontology (AO) [18] has been implied as a means to provide formalized accounts of claims and their supporting background conceptualized as annotations and document parts, respectively. While it is possible in this way to relate individual ontology terms to parts of documents, the AO semantics and use cases suggest that its main application area is representation and support of annotations of documents rather than representation and evaluation of extensive, possibly nested, networks of claims. Nanopublications have been proposed as a container format to encode and publish individual assertions using Semantic Web and Linked Data principles [36]. Ideas to include basic evidence-related information such as references to an information source or a research technique in the provenance portion of a nanopublication have been sketched [20] but appear not to be formally defined as part of a normative specification. The recently drafted micropublications model [37] shares scope with SEE with regard to the representation of extensively justified claims. The models use structurally different conceptualizations which lead to different design patterns for representing claims, their provenance, and the evidence for a claim (see additional file 6).

The central focus of the SEE approach is to provide a formalized account of evidence as claims, their subjects and their argumentative structure using clearly defined concepts and semantics. From this perspective SEE and related works may be seen as complementary: The basic notion of a report part in RDO could be complemented with AO's rich set of selectors as pointers to various elements of scientific reports. Recent alignment of the Citation Typing Ontology (CiTO) and SWAN produced a consolidated set of constructs to characterise bibliographic references [38]. Potentially, nanopublications could be enhanced by using SEE as a model for representing evidence. Also, exploring links of RDO constructs to upper-level ontologies such as SIO [39] or BFO [40] might be beneficial for integration of SEE with other biomedical ontologies.

Claims made in scientific research communications are usually embedded in a contextually and rhetorically rich narrative and attenuated by expressions of epistemic modality [41]. Adding such assertions of certainty to the representation will be an important extension of the SEE approach. Likewise, definition of domain- and application-specific patterns of relevant claims and arguments will be of great value for streamlining the generation of formalized evidence accounts as well as for leveraging text mining approaches for computational identification and extraction of claim subjects and argumentative structure.

## Conclusions

SEE (**S**emantic **E**videnc**E**) is an approach to represent scientific evidence in terms of the argumentative structure of the supporting background. The presented case study suggests that SEE is capable to provide a computationally accessible account of evidence even in complex settings. SEE enables a coherent representation of evidence-related information such as the materials, methods, assumptions, reasoning and information sources used to establish a scientific finding by adopting a consistently claim-based perspective on scientific results. SEE allows for extensible evidence representations, in which the level of detail can be chosen as needed and existing accounts can be extended. Its design permits representation of arbitrary many layers of consecutive interpretation and attribution as well as different evaluations of the same data. SEE is specified as an RDF/OWL based approach for integration with other semantic web resources and linked open data environments but its underlying model can also be used in other knowledge engineering and knowledge representation approaches.

## Availability

Project website: https://purl.org/see.

The latest version of RDO can be accessed from the project website and directly at http://www.purl.org/see/rdo.

## Methods

Protégé 4 [42] was used as ontology engineering environment for developing and testing RDO. Named graph representations were prepared according to the TriG specification [21]. Resources used to represent the case study entities were defined in the http://purl.org/see/gsexample# namespace to provide a homogeneous representation layer independent of mappings to other ontologies or resources. Mappings of individual constructs to other biomedical ontologies were researched using Ontobee [43] and are listed in additional file 4. Representation of biochemical entities (e.g. GS-enzyme, GS-activity) follows the approach described in [44]. The representation of intermediate steps of the investigation described in Tate et al. [29] uses in part design patterns adapted from patterns originally specified for the Ontology for Biomedical Investigations (OBI) [45-47].

We have adopted the following nomenclature for labelling proposition and assertion instances. Labels of proposition instances are derived from the labels of the resources used in its associated graph representation and the type of axiom involved. For restrictions, subclass axioms and type declarations the corresponding keywords of the Manchester OWL Syntax [48] are used. Proposition instances whose graph representation is derived from formalizing a statement of the form "some A related to some B" i.e. consists of the instantiation of a class (A and *related_to *some B) are alternatively labelled as "some A *related_to *some B". Assertion instance labels are specified on the basis of the labels of corresponding proposition instances (linked via *rdo:asserts*) and agents (linked via *rdo:is_assertion_made_by*). If "P" is the proposition label and "A" is the agent label, the assertion is labelled as "! P ! A". Multiple propositions are concatenated by " AND ". Multiple agents are separated by a space symbol. For our case study, author names are further abbreviated by their initials. The rationale of this nomenclature is that it allows to quickly understand *what *is asserted and *who *asserts, maintains a clear connection between assertion and proposition, maintains a connection to the formal representation of the assertion subject and singles out assertions as being labelled with a leading exclamation mark.

## Competing interests

The authors declare that they have no competing interests.

## Authors' contributions

CB and MW conceived the conceptual elements of the SEE approach and developed the abstract model underlying the RDO. CB implemented the RDO in OWL, developed the design patterns and formulated the test case representation with contributions by MW. CB, MW and HGH evaluated several development versions of the design patterns and resulting representations. HGH supervised the research. CB drafted the manuscript. All authors read and approved the final manuscript.

## Supplementary Material

Additional file 1 **rdo_owl.txt**. Full, formal specification of all RDO constructs. This version is also available at http://purl.org/see/rdo/1.0. The latest version of RDO is available at http://purl.org/see/rdo. Click here for file

Additional file 2 **gsexample_owl.txt**. Representation of the argumentative structure and axioms involving the entities used to model the case study in OWL format. Click here for file

Additional file 3 **gsexample_propositions_trig.txt**. Named graph representations of assertion subjects. Click here for file

Additional file 5 **gsexample_sparql.txt**. SPARQL queries for the case study representation. Click here for file

Additional file 6 **SEE_MP_comparison_note.pdf**. A short comparison of central concepts in the SEE and MP (Micropublications) models. Click here for file

Additional file 4 **mappings.pdf**. Mappings of constructs in the case study representation to other biomedical ontologies. Click here for file

## Declarations

Publication of this article was funded by the Virtual Liver project of the German Federal Ministry of Education and Research, grant no. 0315741.

This article has been published as part of *Journal of Biomedical Semantics *Volume 5 Supplement 1, 2014: Proceedings of the Bio-Ontologies Special Interest Group 2013. The full contents of the supplement are available online at http://www.jbiomedsem.com/supplements/5/S1.
