# Supplementary material for: SEE: structured representation of scientific evidence in the biomedical domain using Semantic Web techniques
Source: J Biomed Semantics. 2014 Jun 3;5(Suppl 1):S1. doi: 10.1186/2041-1480-5-S1-S1 (PMC4108886; doi:10.1186/2041-1480-5-S1-S1)
Supplement: Additional file 6 — SEE_MP_comparison_note.pdf. A short comparison of central concepts in the SEE and MP (Micropublications) models. [file 2041-1480-5-S1-S1-S6.pdf]

Central to SEE is the distinction of three complementary entity types: a claim as such (made by a particular agent at a particular occasion, class `rdo:assertion`), its subject (class `rdo:proposition`) and the linguistic form in which this subject is originally communicated (class `rdo:text`). The micropublications model defines a claim as the "principal statement argued for by a micropublication", a statement as a "declarative sentence" and a sentence as a "meaningful symbolic representation". Claims, statements and sentences in the micropublications model form a subclass hierarchy under the concept of 'representation'. The predicate *supports* is the principal relation in the micropublications model to relate two representations, i.e. claims, statements, references, attributions, data (subproperty *dataSupports*) and methods (subproperty *methodSupports*). In SEE due to the complementary nature of assertion, proposition and text, complementary relations are used to link assertions to propositions, texts, agents and reports. In SEE, assertions, are only ever supported by other assertions (via *`rdo:is_inferred_from`*). Datasets or methods are referenced via the graph representation of an assertion's subject and reports and agents via its provenance.

These are structurally different conceptualizations. They lead to different design patterns for what is a common aim in SEE and in micropublications: representing claims, their provenance and the evidence for them.
