# Supplementary material for: SEE: structured representation of scientific evidence in the biomedical domain using Semantic Web techniques
Source: J Biomed Semantics. 2014 Jun 3;5(Suppl 1):S1. doi: 10.1186/2041-1480-5-S1-S1 (PMC4108886; doi:10.1186/2041-1480-5-S1-S1)
Supplement: Additional file 4 — mappings.pdf. Mappings of constructs in the case study representation to other biomedical ontologies. [file 2041-1480-5-S1-S1-S4.pdf]

Resources mappings

Mappings were only considered when documentation in source ontologies was sufficient to establish that the concepts could be used at least in some contexts interchangeably. Resources used to represent the test case study not included in this table could not be mapped.

GS construct	IRI	IRI mapped	label
agent	http://purl.org/see/rdo#agent	http://www.w3.org/ns/prov#Agent	agent
assay	http://purl.org/see/gsexample#assay	http://purl.obolibrary.org/obo/OBI_0000070	assay
data item	http://purl.org/see/gsexample#data_item	http://purl.obolibrary.org/obo/IAO_0000027	data item
GS-enzyme	http://purl.org/see/gsexample#gs_enzyme	http://purl.obolibrary.org/obo/PR_000008058	glutamine synthetase
person	http://purl.org/see/rdo#person	http://www.w3.org/ns/prov#Person	Person
report	http://purl.org/see/rdo#report	http://purl.obolibrary.org/obo/IAO_0000088	report
report part	http://purl.org/see/rdo#report_part	http://purl.obolibrary.org/obo/IAO_0000091	obsolete_report element
sample	http://purl.org/see/gsexample#sample	http://purl.obolibrary.org/obo/OBI_0100051	specimen
text	http://purl.org/see/rdo#text	http://purl.obolibrary.org/obo/IAO_0000300	textual entity
achieves objective	http://purl.org/see/gsexample#achieves_objective	http://purl.obolibrary.org/obo/OBI_0000417	achieves_planned_objective
bears	http://purl.org/see/gsexample#bears	http://purl.obolibrary.org/obo/BFO_0000053	is bearer of
has input	http://purl.org/see/gsexample#has_input	http://purl.obolibrary.org/obo/OBI_0000293	has_specified_input
is input of	http://purl.org/see/gsexample#is_input_of	http://purl.obolibrary.org/obo/OBI_0000295	is_specified_input_of
has output	http://purl.org/see/gsexample#has_output	http://purl.obolibrary.org/obo/OBI_0000299	has_specified_output
is output of	http://purl.org/see/gsexample#is_output_of	http://purl.obolibrary.org/obo/OBI_0000312	is_specified_output_of
has author	http://purl.org/see/rdo#has_author	http://purl.org/dc/elements/1.1/creator	Creator
		http://purl.org/pav/authoredBy	authored by
measurement of	http://purl.org/see/gsexample#measurement_of	http://purl.obolibrary.org/obo/IAO_0000221	is quality measurement of
realizes	http://purl.org/see/rdo#realizes	http://purl.obolibrary.org/obo/BFO_0000055	realizes
